# Mandibular fracture due to rare manifestation of Langerhans cell histiocytosis

**DOI:** 10.5935/0103-507X.20190048

**Published:** 2019

**Authors:** Fábio Vieira de Miranda, William Phillip Pereira da Silva, Renato Victor de Oliveira, Gustavo Antônio Correia Momesso, Tárik Ocon Braga Polo, Leonardo Pérez Faverani

**Affiliations:** 1 Divisão de Cirurgia Oral e Maxilofacial, Departamento de Cirurgia e Clínica Integrada, Faculdade de Odontologia de Araçatuba, Universidade Estadual Paulista Júlio de Mesquita Filho - Araçatuba (SP), Brasil.; 2 Departamento de Cirurgia, Faculdade de Odontologia, Universidade de Maringá - Maringá (PR), Brasil.

**Dear Editor,**

Langerhans cell histiocytosis (LCH) is reported as being rare in the literature, with an incidence of 5 cases per 1 million per year and a prevalence of 3.7:1 in men:women. LCH is generally limited to an organ, most commonly lesions of the bone, whether solitary or multiple, that tend to appear in the cranial or femoral region in children younger than 10 years and in the costal arches, scapula or mandible in patients up to 20 years of age.^(1-3)^ The diagnosis is based on clinical symptoms, radiographic symptoms and especially histological examination after biopsy.

**Figure 1:**
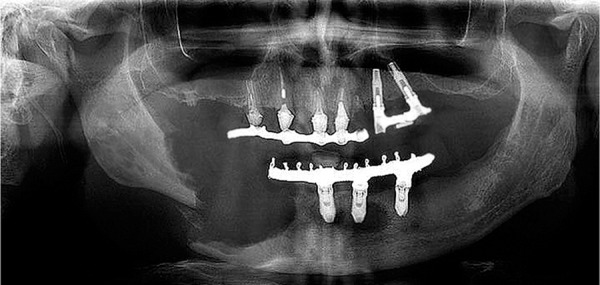
Panoramic radiography presenting a pathological fracture of the mandible on the right side.

**Figure 2:**
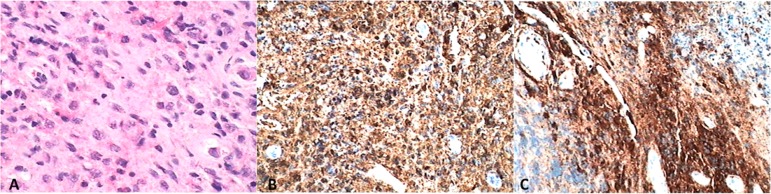
Histological exam. (A) Eosin and hematoxylin; (B-C) Positive reaction to immunohistochemistry by CD-1a, CD207 (Langerin) and Protein S100.

We present a rare manifestation in a male patient, 51 years old, with clinical and radiographic examinations presenting a pathological fracture of the mandible on the right side (Figure 1), due to a radiolucent lesion of approximately 2.5cm, that was circumscribed and well delimited, and the presence of another lesion in the symphysis region that was approximately 0.5cm in diameter. The histological finding was consistent with the infiltration of Langerhans cells, macrophages, lymphocytes, eosinophils, granulocytes and giant cells, that positively reacted to immunohistochemistry for CD-1a, CD207 (Langerin) and Protein S100 (Figure 2A - C).^(4,5)^

The rapid evolution and aggressiveness of the lesion, as well as the initial diagnostic hypothesis of being a malignant lesion, indicated that treatment should involve hospital admission and multidisciplinary planning, which increases the time of treatment and costs because the patient will need reconstructive surgery to restore his functional and aesthetic quality of life.

*Fábio Vieira de Miranda Division of Oral and Maxillofacial Surgery, Department of Surgery and Integrated Clinic, Faculdade de Odontologia de Araçatuba, Universidade Estadual Paulista Júlio de Mesquita Filho - Araçatuba (SP), Brazil.* *William Phillip Pereira da Silva Division of Oral and Maxillofacial Surgery, Department of Surgery and Integrated Clinic, Faculdade de Odontologia de Araçatuba, Universidade Estadual Paulista Júlio de Mesquita Filho - Araçatuba (SP), Brazil.* *Renato Victor de Oliveira Department of Surgery, Faculdade de Odontologia, Universidade de Maringá - Maringá (PR), Brasil.* *Gustavo Antônio Correia Momesso Division of Oral and Maxillofacial Surgery, Department of Surgery and Integrated Clinic, Faculdade de Odontologia de Araçatuba, Universidade Estadual Paulista Júlio de Mesquita Filho - Araçatuba (SP), Brazil.* *Tárik Ocon Braga Polo Division of Oral and Maxillofacial Surgery, Department of Surgery and Integrated Clinic, Faculdade de Odontologia de Araçatuba, Universidade Estadual Paulista Júlio de Mesquita Filho - Araçatuba (SP), Brazil.* *Leonardo Pérez Faverani Division of Oral and Maxillofacial Surgery, Department of Surgery and Integrated Clinic, Faculdade de Odontologia de Araçatuba, Universidade Estadual Paulista Júlio de Mesquita Filho - Araçatuba (SP), Brazil.*
